# Management and obstetric outcomes of 17 heterotopic interstitial pregnancies

**DOI:** 10.1186/s12884-018-1700-x

**Published:** 2018-03-27

**Authors:** Yuan Jiang, Jie Chen, Huaijun Zhou, Mingming Zheng, Ke Han, Jingxian Ling, Xianghong Zhu, Xiaoqiu Tang, Rong Li, Ying Hong

**Affiliations:** 1 Department of Obstetrics and Gynecology, Nanjing Drum Tower Hospital, Nanjing Medical University, Nanjing, 210008 China; 2 Department of Obstetrics and Gynecology, Nanjing Drum Tower Hospital, Affiliated Hospital of Nanjing University Medical School, Nanjing, 210008 China

**Keywords:** Heterotopic interstitial pregnancy, Laparoscopic cornual resection, Selective embryo reduction, Expectant management

## Abstract

**Background:**

Heterotopic interstitial pregnancy is a rare variant of heterotopic pregnancies, and it poses challenges in treating the heterotopic pregnancy and preserving the intrauterine pregnancy. However, there is no clear consensus regarding the optimal management. The aim of this study was to investigate the pregnancy outcomes of women diagnosed with heterotopic interstitial pregnancy.

**Methods:**

A total of 17 women diagnosed with heterotopic interstitial pregnancy between July 2010 and December 2015 were included. General characteristics of each patient, including age, gravidity and parity, history of pelvic inflammatory disease or surgery, and especially the corresponding therapeutic interventions, were retrospectively analyzed. Moreover, pregnancy outcomes were further followed by face-to-face interview.

**Results:**

Of the 17 patients, 10 (58.5%) underwent surgical treatment (7 laparoscopic cornual resection, and 3 laparotomy); and 3 cases simultaneously terminated the intrauterine pregnancy by suction evacuation. Compared with laparotomy, laparoscopic cornual section showed shorter operative time (median 40 vs. 70 min), less blood loss (150 vs. 400 ml) and shorter hospital stay (2 vs. 4 days). In addition, 4 (23.5%) patients underwent selective embryo reduction under transvaginal ultrasound guidance. Expectant management was chosen in the remaining 3 patients. In the follow-up study, other than a case of missed miscarriage, the other 13 women who remained committed to their pregnancies all delivered healthy babies either by caesarean section or vaginal birth. No congenital anomalies were reported, and all the infants were in good growth and development.

**Conclusions:**

Laparoscopic cornual resection is a feasible approach with favorable surgical and long-term pregnancy outcomes. Additionally, medical or expectant management may be a viable treatment option for selected symptom-free patient. Although the survival of the intrauterine pregnancy could not always be assured, the prognosis for a woman with heterotopic interstitial pregnancy is generally good.

## Background

Heterotopic interstitial pregnancy, a rare form of pregnancy that involves the coexistence of an ectopic interstitial pregnancy and intrauterine pregnancy, is one of the most life-threatening types of all the ectopic gestations [1]. The calculated incidence is documented to be as high as 1/3600 in pregnancies after assisted reproductive technology (ART) [2].

However, most of these women were asymptomatic or only showed non-specific symptoms such as abdominal pain and vaginal bleeding [3]. The clinicians might not consider the possibility of extrauterine pregnancy in the case of a confirmed intrauterine gestation by ultrasonography. Therefore, the diagnosis of heterotopic interstitial pregnancy is often delayed, even after its rupture [2] and meticulous care is required, especially in the patients with relevant risk factors associated with heterotopic pregnancy such as previous history of ectopic pregnancy, pelvic surgery, or pelvic inflammatory diseases [2, 4].

The treatment of heterotopic interstitial pregnancy aims to preserve the intrauterine pregnancy while removing or interrupting the evolution of the ectopic interstitial pregnancy using a minimally invasive method. The therapeutic modalities include surgical, medical or expectant treatment [2]. However; there is no consensus for the treatment of heterotopic interstitial pregnancy. Therefore, in the present study, we aimed to investigate the therapeutic interventions and corresponding obstetric outcomes of women diagnosed with heterotopic interstitial pregnancy.

## Methods

### Study population

We retrospectively analyzed all the inpatients in the department of obstetrics and gynecology from Nanjing Drum Tower Hospital between July 2010 and December 2015. A total of 17 patients with diagnosed heterotopic interstitial pregnancy were included in the present study, suggesting an overall incidence of 0.04% for all the deliveries (*n* = 40,761) at our institution over the same study period. The Reproductive medicine center of our hospital is the first batch approved by the Ministry of health to carry out ART, and therefore it serves for infertility patients throughout the country. Nearly 18,000 patients conceived by ART during the study period. The heterotopic interstitial pregnancy was diagnosed by experienced radiologist using 2-D ultrasound.

The criteria included an intrauterine pregnancy along with feature of a co-exisiting interstitial pregnancy, i.e. a gestational sac visualized high in the fundus, and not surrounded by 5 mm of myometrium in all planes; and a gestational sac seen separately and < 1 cm from the most lateral edge of the uterine cavity [5]. Exclusion criteria referred to an ectopic gestational sac observed in another location such as fallopian tube, ovary, cervix, or abdominal cavity. This study was performed according to the Declaration of Helsinki and approved by the institutional review boards of Nanjing Drum Tower Hospital (NJDTH20160810).

### Data collection

Each patient’s age, gravidity and parity, history of pelvic inflammatory disease or surgery, gestational weeks at diagnosis, and clinical symptoms of abdominal pain and vaginal bleeding was retrospectively collected by reviewing the medical records. We also recorded whether the current pregnancy had been conceived naturally or by ART. The gestational age was calculated by adding 14 days to the date of embryo transfer. Routine transvaginal ultrasound examination was performed at approximately 6 weeks of gestation (i.e. 4 weeks after embryo transfer) in all the pregnant women, and a repeat ultrasound scan was performed after a two weeks interval, or promptly if the patient presented with clinical symptoms of abdominal pain or vaginal bleeding at any time.

The therapeutic intervention, including surgery (either laparoscopy (KARL STORZ ENDOSKOPE) or laparotomy with cornual resection, or hysterectomy), medical treatment, or expectant management, was collected from each patient. The operative time, volume of intra-operative haemorrhage, length of hospital stay and incidence of intra-operative and postoperative complications were recorded. A transvaginal ultrasound scan was performed on the third day postoperative or before discharge to confirm the fetal viability of intrauterine gestation in each patient who continued with their intrauterine pregnancy.

Medical treatment comprised aspiration of ectopic fetal heart along with products of conception and local injection of 10% potassium chloride (KCl) into cornual gestational sac under transvaginal ultrasound guidance (SIEMENS G50). In the patients under medical or expectant management, due to the possibility of miscarriage of intrauterine pregnancy and rupture of extrauterine pregnancy, repeated clinical and ultrasound examinations were performed weekly until a complete resolution of interstitial pregnancy was confirmed.

Furthermore, to investigate the pregnancy outcome, we invited each patient to a face-to-face interview conducted during August 2016 to January 2017. Of the 17 patients, 16 women gave their oral and written informed consent on behalf of themselves and their children during the face-to-face interview. Nevertheless, one case refused our face-to-face invitation over the telephone because she underwent a missed miscarriage at 8 gestational weeks following expectant management; but she provided the verbal informed consent for participation in the study and publication, and also agreed to sign the written informed consent if requested. The median child’s age was 3.5 years (range, 1–6 years) at our follow-up study. Data recorded for all the live births comprised of gestational age, mode of delivery, the infant’s birth weight, height and gender, which were collected from the child health record. This parent held record contained details of the child’s vaccinations, growth, and development, which was assessed by professional child health care doctor. During the face-to-face interview, based on the child health and development record, we evaluated the infant’s overall health condition including congenital malformations, intelligence, hearing, and language. Each infant’s height and weight was measured.

### Statistical analysis

Statistical analysis was performed with the SPSS software (SPSS Standard version 17.0, SPSS Inc., Chicago, IL). Quantitative data non-normally distributed were presented as median (range) and compared by Wilcoxon rank sum test between two groups; categorical variables were reported as number (percentage). A two-sided *P* value< 0.05 is considered statistically significant.

## Results

From July 2010 through December 2015, a total of 17 inpatients were diagnosed with heterotopic interstitial pregnancy in our institution. As shown in Table 1, all 17 women achieved the current pregnancies by in-vitro fertilization and embryo transfer. The median mother’s age and gestational age at diagnosis was 29 years (range, 24–35 years) and 7 weeks (range, 6–14 weeks), respectively. None of the pregnant women were multiparous. Of the 17 patients, 13 (76.5%) had previously undergone at least one pelvic surgical intervention such as salpingectomy, hystero-laparoscopy, and pelvic adhesiolysis; and 6 (35.3%) patients had previously undergone either unilateral or bilateral salpingectomy for ectopic pregnancy (Table 1). The clinical manifestations of abdominal pain or vaginal bleeding were observed in 8 (47.1%) of the 17 cases, but neither hypovolaemic shock nor maternal death was reported due to timely diagnosis and treatment. However, the remaining 9 asymptomatic patients were diagnosed incidentally on routine ultrasound examination after ART procedure.

**Table 1:** Characteristics and pregnancy outcomes of the patients diagnosed with heterotopic interstitial pregnancy (*n* = 17)

No.	Age (years)	Gravidity/parity	Risk factors	Gestational age (week + days)	Symptoms	Interstitial gestation	Treatment	Pregnancy Outcome
1	30	5/0	IVF-ET	6	Asymptomatic	Fetal cardiac activity	Explo-left cornual resection + suction evacuation	NA
2	25	1/0	IVF-ET, LSC-RS, LTL	6	Vaginal bleeding	Fetal cardiac activity	Same as above	NA
3	34	4/0	IVF-ET, LSC-RS, LTL (Ectopic pregnacy)	6 + 2	Abdominal pain	Yolk sac (20*23*19 mm)	LSC-right cornual resection + suction evacuation	NA^a^
4	28	2/0	IVF-ET, LSC-RS (Ectopic pregnancy)	6	Asymptomatic	Fetal cardiac activity	LSC-right cornual resection	CS at 35 + 4 weeks due to preterm labor, female, 2305 g
5	28	1/0	IVF-ET, LSC-BS	7 + 6	Asymptomatic	Fetal cardiac activity	Explo-right cornual resection	CS at 38 weeks, male, 3540 g
6	27	2/0	IVF-ET, LSC-BS	6	Abdominal pain	Yolk sac (16*19*16 mm)	LSC-left cornual resection	CS at 38 + 1 weeks, male, 3020 g
7	35	3/0	IVF-ET, LSC-LS (Ectopic pregnancy)	8	Asymptomatic	Yolk sac (21*22*23 mm)	LSC-left cornual resection	CS at 38 + 2 weeks, male, 2980 g
8	30	3/0	IVF-ET, LSC-RS (Ectopic pregnancy)	7 + 5	Vaginal bleeding	Yolk sac (17*16*16 mm)	LSC-left cornual resection	CS at 38 + 4 weeks, male, 3480 g
9	32	1/0	IVF-ET	12	Vaginal bleeding	Yolk sac (41*38*43 mm)	LSC-right cornual resection	CS at 39 weeks, female,3100 g
10	29	3/0	IVF-ET	6 + 5	Asymptomatic	Fetal cardiac activity	LSC-right cornual resection	CS at 39 + 1 weeks, female,3460 g
11	24	1/0	IVF-ET, LSC-RS, Pelvic adhesiolysis	6 + 5	Asymptomatic	Fetal cardiac activity	cornual embryo reduction	CS at 38 weeks, male, 2670 g
12	28	3/0	IVF-ET, LSC-BS (Ectopic pregnacy)	6 + 1	Abdominal pain	Fetal cardiac activity	cornual embryo reduction	CS at 38 + 5 weeks, female, 2800 g
13	26	1/0	IVF-ET	6 + 3	Asymptomatic	Fetal cardiac activity	cornual embryo reduction	CS at 39 weeks, female, 2730 g
14	33	2/0	IVF-ET, LSC	7	Asymptomatic	Fetal cardiac activity	cornual embryo reduction	CS at 39 + 2 weeks, male, 3020 g
15	33	3/0	IVF-ET, LSC-RS (Ectopic pregnancy)	6	Vaginal bleeding	Yolk sac (8*9*9 mm)	Conservative	Missed miscarriage at 8 weeks
16	34	3/0	IVF-ET, LSC-BS	14	Asymptomatic	Yolk sac (25*23*23 mm)	Conservative	CS at 38 + 2 weeks, female, 2940 g
17	27	1/0	IVF-ET, H-LSC	6 + 4	Abdominal pain	Yolk sac (20*19*19 mm)	Conservative	VD at 35 + 6 weeks due to PROM, male, 2230g^b^

*LSC* laparoscopy, *LTL* left tubal ligation, *LSC-RS* laparoscopic right salpingectomy, *LSC-BS* laparoscopic bilateral salpingectomy, *H-LSC* hystero-laparoscopy, *LSC-LS* laparoscopic left salpingectomy, *Explo*- exploratory, *NA* not available, *PROM* premature rupture of membrane

^a^The woman conceived again by ART procedure; and it was confirmed to be normal intrauterine gestation of 15 weeks in the telephone follow-up study

^b^She refused emergency cesarean section but attempted vaginal delivery because the cervix was 3 cm wide when seeing the doctor

Of the total 17 patients, 10 (58.5%) underwent surgical treatment (7 laparoscopic cornual resection, and 3 laparotomy). Laparotomy in those three patients was mainly attributed to poor family economic situation or restriction of laparoscopic operation on weekends or holidays in our institution. However, three cases simultaneously terminated the intrauterine pregnancy by suction evacuation, as they were aware of the risk of uterine rupture later in pregnancy. The median operative time was 45 min (range, 28–135 min) and estimated blood loss in the operation was 175 ml (range, 20–750 ml). Only one case had blood transfusion (2 units of red blood cells) after the operation. The median length of postoperative hospital stay was 2.5 days (range 2–7 days) without any intraoperative and postoperative complications such as miscarriage or uterine rupture. Furthermore, compared with laparotomy, the laparoscopic treatment showed shorter operative time (median 40 vs. 70 min), less blood loss in the operation (150 vs. 400 ml) and shorter hospital stay (2 vs. 4 days), although the difference was not statistically significant (*P* = 0.11, 0.11 and 0.09 respectively). It was likely due to the small sample size of the study.

After fully informed consent, 4 (23.5%) patients (including 3 with no clinical symptoms and 1 with mild abdominal pain) underwent selective embryo reduction, i.e. aspiration of the ectopic fetal heart and local injection of 10% KCl into the interstitial pregnancy sac under direct transvaginal ultrasound guidance. Expectant management was chosen in the remaining 3 (17.6%) patients with no symptoms or only a small amount of vaginal bleeding (Table 1). Importantly, only a crown rump length or yolk sac was shown in either case, but no fetal cardiac activity in ectopic interstitial gestation was seen by ultrasound scan. All of these 7 patients were followed up closely by weekly clinical and ultrasound assessment. Unfortunately, missed miscarriage of intrauterine gestation at 8 gestational weeks was confirmed in one case with expectant management. However, no surgical treatment was indicated due to persistent existence of ectopic cardiac activity or rupture of interstitial gestation in the other six patients.

In the follow-up study, the three pregnant women, who had simultaneously terminated intrauterine pregnancy, all recovered well after the surgery. In addition, as shown in Table 1, only one of the 14 cases who had an ongoing intrauterine pregnancy was diagnosed with a missed miscarriage at 8 gestational weeks; but the other 13 patients all delivered healthy live babies vaginally or by cesarean section. However, only one case attempted vaginal delivery because the dilatation of cervix was 3 cm when seeing the doctor. The other 12 women all delivered their babies by elective cesarean section due to fears of uterine rupture or cultural factors. It was worthy that no congenital anomalies were reported, and all the infants were in normal growth and development.

## Discussion

The incidence of heterotopic interstitial pregnancy has considerably increased, with the widespread use of assisted reproductive technology and the rising frequency of tubal and pelvic inflammatory diseases. However, the optimal management of heterotopic interstitial pregnancy still remains controversial. There are currently limited clinical data or only several case reports in the literature, especially in developing countries. In the present study, we conducted a retrospective review of the therapeutic interventions and further investigated the corresponding obstetric outcomes of all the 17 patients diagnosed with heterotopic interstitial pregnancy over the past five years in our centre.

Previous history of pelvic inflammatory disease, pelvic surgery, and ART are associated with the incidence of heterotopic interstitial pregnancy [2]. Consistent with these studies, we found that all of the 17 pregnant women had been conceived through ART, and 76.5% (13/17) had a history of ectopic pregnancy, pelvic inflammatory disease or surgery. In the present study, neither hypovolemic shock nor maternal death was reported. It seemed that this was not a typical population presenting with ectopic pregnancy. We speculated that it may be related to the fact that all these patients after embryo transfer were provided with detailed health education on red flag symptoms of ectopic pregnancy; and therefore they sought medical advice and received timely professional assessment and treatment.

The feasibility of surgical cornual resection has been demonstrated in treating heterotopic interstitial pregnancy. However, most of these studies were only case reports and mainly conducted in the developed countries or regions [3, 6–10]. Some authors indicated that the long operative time and altered intraperitoneal carbon dioxide environment throughout the laparoscopic operation may involve surgical and anesthetic risk, causing an adverse effect on maternal morbidity and the surviving intrauterine pregnancy [10]. In the present study, we found that all the infants born to the seven pregnant women, who had undergone laparoscopic cornual resection or laparotomy, were in good health and no congenital conformations were reported. In addition, compared with laparotomy, laparoscopic treatment showed the shorter hospital stay, fewer surgical wounds, and reduced use of antibiotics and analgesics. Therefore, laparoscopic cornual resection appears to be a safe and viable treatment option and should be considered, especially in the patients without signs of cornual rupture [11]. The other three women had selected laparotomy only because of poor family economic situation or restriction of laparoscopic operation at night time, weekends or holidays in our institution. We hope that improved access to laparoscopic treatment can be offered in the future.

Surgical cornual resection may increase the risk of delayed haemorrhage and uterine rupture. Compared with surgical treatment, the medical approach is less expensive, less invasive with minimal blood loss and quicker recovery. Therefore, medical embryo reduction of interstitial gestation has been recommended in the patient with hemodynamically stable status [12, 13]. It consists of injecting methotrexate, prostaglandin, or KCL directly into ectopic gestational sac under the guidance of transvaginal ultrasonography. Kim et al. suggested that KCl has reportedly been associated with intrauterine damage, periventricular leukomalacia, and limb anomalies [3]. However, more studies confirmed that no teratogenicity secondary to local injection of KCl was reported [14]. Therefore, selective embryo reduction using local injection of 10% KCl has been performed in four patients after fully informed consent at our centre. To ascertain whether KCl may have adverse effects on the pregnancy outcomes, we further evaluated the infant’s overall health condition during face-to-face interview. No congenital malformation was found, and all the infants were in normal growth and development. Therefore, it indicated that selective embryo reductionby local injection of KCl during the first trimester might be a viable treatment approach.

Expectant management seems to offer a third possible and safe therapeutic alternative [2]. Nevertheless, the current reported clinical studies were limited [15, 16]. In the present study, expectant management had been chosen in three patients (Table 1). Other than a single case of missed miscarriage, an uneventful ongoing pregnancy was documented in the other two cases. Therefore, expectant management might be a viable alternative for the interstitial pregnancy with absence of fetal cardiac activity and limited gestational sac size. Nevertheless, because of the potential risk of continued growth of interstitial gestation and subsequent rupture, cornual gestation and subsequent rupture, serial ultrasound scans and close clinical assessment are necessary [2].

## Conclusion

In summary, laparoscopic cornual resection is a feasible approach with favorable surgical and long-term pregnant outcomes. Medical or expectant management may be an efficient treatment alternative for selected symptom-free patient. Although the survival of the intrauterine pregnancy could not always be assured, the prognosis for a woman with heterotopic interstitial pregnancy is generally good.
